# A 3D-Printed PMMA Microneedle-Based TSA-ELISA Platform for Noninvasive Inflammatory Biomarker Detection

**DOI:** 10.3390/mi16111286

**Published:** 2025-11-14

**Authors:** Minghui Xu, Qingyu Ruan, Yukun Ren

**Affiliations:** 1 School of Mechatronics Engineering, Harbin Institute of Technology, Harbin 150001, China; 2 State Key Laboratory of Robotics and System, Harbin Institute of Technology, Harbin 150001, China

**Keywords:** microneedles, biosensing platform, inflammatory biomarkers, noninvasive detection

## Abstract

Inflammatory cytokines and proteins are essential indicators of immune status and disease progression; however, conventional assays rely on invasive sampling and complex processing, restricting their use in real-time monitoring. Here, we present a 3D-printed poly(methyl methacrylate) (PMMA) microneedle-based biosensing platform integrated with a tyramide signal amplification–enhanced enzyme-linked immunosorbent assay (TSA–ELISA) for noninvasive and highly sensitive detection of inflammatory biomarkers in interstitial fluid. The microneedles exhibit precise geometry, adequate mechanical strength, and excellent biocompatibility, facilitating efficient skin penetration and biomarker capture. Stepwise chemical functionalization ensured stable antibody immobilization, while TSA significantly amplified detection signals. The platform achieved reliable, reproducible, and multiplex detection of cytokines and albumin in both healthy individuals and patients with inflammatory skin conditions. Notably, the measured cytokine level in lesional skin of eczema patients was 97.7 pg/mL, showing a significant difference from the 62.8 pg/mL observed in healthy subjects. This MN-based TSA–ELISA system offers a robust and minimally invasive strategy for monitoring inflammation-related biomarkers, holding great potential for clinical diagnostics and personalized healthcare applications.

## 1. Introduction

Wearable biosensing platforms have recently emerged as powerful tools for real-time, minimally invasive monitoring of physiological states, offering new opportunities for continuous health monitoring, early disease detection, and personalized medical management [1,2]. Among these, microneedle (MN)-based systems are particularly attractive because they can painlessly access interstitial fluid (ISF), which closely mirrors blood composition, without the need for conventional invasive sampling [3,4]. MNs can function as both sampling and sensing devices, enabling dynamic, in situ monitoring of diverse biomarkers. Recent advances have demonstrated the integration of MNs into wearable formats, allowing continuous tracking of glucose, lactate, and inflammatory cytokines, and highlighting their potential for point-of-care diagnostics and personalized health applications [5,6].

Inflammation plays a pivotal role in the onset and progression of various pathological conditions, including autoimmune disorders, cancers, and infectious diseases [7,8,9]. Dynamic fluctuations of cytokines and other inflammatory proteins provide critical insights into immune status and disease progression, emphasizing the importance of sensitive and accurate biomarker detection [10]. Conventional techniques such as enzyme-linked immunosorbent assays (ELISAs) exhibit high sensitivity and specificity, yet they depend on invasive blood sampling, intricate sample preparation, and lengthy analytical processes, limiting their practicality for rapid or point-of-care applications [11,12,13]. These challenges underscore the significance of MN-based biosensing platforms, particularly when combined with advanced signal amplification strategies such as tyramide signal amplification (TSA), which enable highly sensitive, noninvasive, and real-time detection of inflammation-related biomarkers.

Recently, microneedle-based biosensing technologies have emerged as a highly promising platform for biomarker detection due to their minimally invasive, painless, and real-time monitoring capabilities [14,15]. These devices have evolved from simple drug delivery tools to integrated diagnostic platforms, encompassing various types including solid MNs for skin pretreatment, coated MNs with surface-bound recognition elements, and hydrogel MNs that swell to enrich biomarkers [16]. MNs can penetrate the stratum corneum to directly access interstitial fluid (ISF), which closely correlates with blood composition, enabling dynamic assessment of physiological states [17,18]. ISF represents a rich source of biomarkers, and MNs offer a minimally invasive alternative to traditional sampling methods like suction blister and microdialysis [19]. Although MNs have been fabricated from polymers, metals, and ceramics, achieving both excellent biocompatibility and sufficient mechanical strength while maintaining precise microstructures remains a significant challenge [20,21].

In recent years, MN-based biosensing systems have advanced rapidly toward continuous and multiplexed health monitoring. Various sensing mechanisms—such as electrochemical, optical, and immunoassay-based detection—have been integrated into MNs for the quantification of diverse analytes including glucose, lactate, cytokines, and nucleic acids. For instance, electrochemical MN sensors enable real-time glucose tracking, while immunoaffinity MNs facilitate direct in situ detection of inflammatory cytokines such as TNF-α and IL-6 from ISF. Hydrogel MNs, with their inherent biocompatibility and swelling properties, have also been employed for passive sampling and signal transduction. Despite these advances, challenges persist in improving mechanical robustness for reliable skin insertion, achieving stable biofunctionalization for reproducible detection, and enhancing analytical sensitivity for trace-level biomarkers in complex biofluids [22,23,24].

Three-dimensional (3D) printing provides a flexible and customizable approach for MN array fabrication, offering high geometric accuracy and reproducibility. Advanced techniques like laser-induced direct writing enable rapid in situ thermoset material curing, allowing freeform 3D structuring without support materials and programmable control over mechanical properties—capabilities highly suited to complex MN sensor fabrication [25]. The materials spectrum for 3D-printed MNs has expanded significantly, encompassing stimuli-responsive DNA hydrogels that enable programmable drug release [26], mechanically robust PMMA for structural integrity, and biocompatible poly(ethylene glycol) diacrylate (PEGDA) for enhanced tissue compatibility. Among candidate materials, poly(methyl methacrylate) (PMMA) stands out for its optical transparency, mechanical robustness, and biocompatibility, making it an ideal substrate for MN platforms [27].

To further enhance detection sensitivity, various signal amplification strategies have been developed, among which tyramide signal amplification (TSA) is particularly notable [28]. TSA relies on horseradish peroxidase (HRP) to catalyze the oxidation of tyramide substrates, generating highly reactive radicals that covalently bind to target molecules or adjacent tyrosine residues [29,30]. This reaction significantly amplifies the detection signal while reducing background noise [31]. When integrated with ELISA, TSA yields a TSA-enhanced enzyme-linked immunosorbent assay (TSA–ELISA), markedly improving sensitivity and quantitative accuracy for target biomolecules [32,33].

In this study, we established a high-sensitivity detection platform utilizing 3D-printed PMMA microneedles for the quantitative analysis of the pro-inflammatory cytokine TNF-α and the serum protein albumin. High-precision, photopolymerized 3D-printed MN arrays exhibit well-defined geometries and sufficient mechanical strength to effectively penetrate the skin barrier and sample biomolecules from ISF. Stepwise chemical functionalization of the MN surface enabled antigen conjugation, constructing an immunoreaction-driven detection interface capable of efficiently capturing target antibodies. To maximize sensitivity, we optimized the concentration of detection antibodies and integrated TSA-based ELISA, resulting in substantial signal enhancement for ultra-sensitive quantitative analysis.

**Figure 1:**
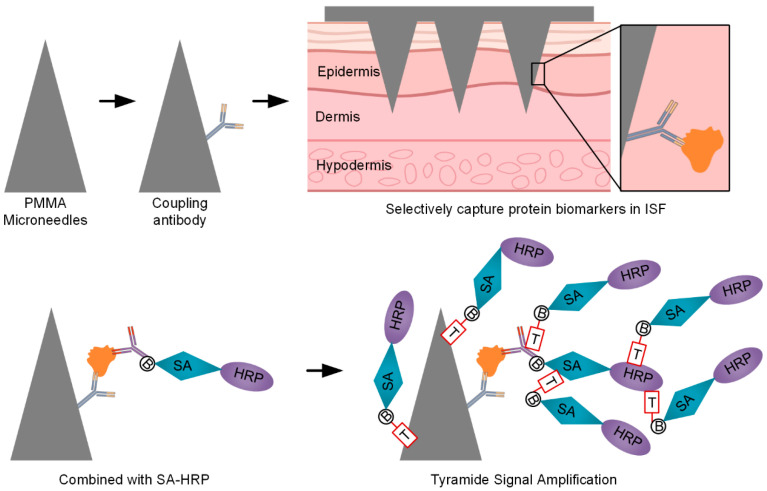
Three-dimensionally printed microneedles with TSA for ultra-sensitive biomarker detection.

Testing in ISF from healthy volunteers and patients with eczema demonstrated that the platform produces stable signals with high reproducibility and clear differentiation between blank controls and target analytes. Further quantification of albumin confirmed the platform’s versatility, indicating that the optimized ELISA system is applicable not only to TNF-α but also to other biomarkers, supporting multi-target, high-sensitivity, and noninvasive analysis. Overall, the results demonstrate that this MN-based TSA–ELISA platform provides a robust and feasible approach for noninvasive, real-time, quantitative detection of inflammation-related biomarkers. As illustrated in Figure 1, this study introduces a novel strategy integrating 3D-printed microneedles, immunosignal amplification, and microneedle-based sensing, providing a promising approach for noninvasive, real-time biomarker monitoring with potential applications in clinical diagnostics and personalized medicine.

## 2. Materials and Methods

### 2.1. Fabrication and Mechanical Characterization of PMMA Microneedles

PMMA microneedle arrays (MNs) were fabricated using a photopolymerization-based 3D printing approach. Photocurable PMMA resin was loaded into a digital light processing 3D printer (BMF, Chongqing). Arrays were designed in a 4 × 4 configuration, with each needle having a height of 1000 µm and a base width of 400 µm. Printing was performed with a 405 nm light source (typical for bottom-up DLP systems) at an irradiance of ~6 mW·cm^−2^. For a layer thickness of 10 µm, the exposure time was set to 2.0 s per layer. To ensure robust adhesion of the first layers to the build plate, the initial 3 base layers were cured with an extended exposure of 45 s each.

After printing, the arrays were gently rinsed with isopropanol for 3 min to remove uncured resin, followed by UV curing for 10 min (wavelength 365 nm) to ensure complete polymerization and structural stability. The morphology and uniformity of the arrays were examined using a stereomicroscope, and representative images were captured.

Mechanical properties were evaluated through axial compression tests using a universal testing machine. Arrays were positioned vertically on the sample stage, with a flat stainless steel plate as the compression surface. Load was applied at a constant rate of 0.1 mm/min, and force–displacement curves were recorded to characterize the mechanical behavior of the microneedles. All tests were performed in triplicate, and the resulting curves were analyzed to assess reproducibility.

### 2.2. Surface Functionalization and TNF-α Capture Antibody Immobilization

A stepwise chemical modification strategy was employed to immobilize Tumor Necrosis Factor-alpha (TNF-α) capture antibodies onto PMMA microneedles. Freshly 3D-printed PMMA MN arrays were first treated with oxygen plasma (100 W, 1 min) using a plasma cleaner (Diener Electronic GmbH, Ebhausen, Germany) to generate hydroxyl groups on the surface, enhancing hydrophilicity and chemical reactivity. The MNs were then immediately immersed in 2% (*v*/*v*) 3-aminopropyltriethoxysilane (APTES) in absolute ethanol at room temperature for 1 h to introduce amino groups, with gentle shaking to ensure uniform coverage [34]. After incubation, MNs were rinsed thoroughly with anhydrous ethanol three times to remove unreacted APTES, and dried under a gentle stream of nitrogen for 10 min.

Aminated MNs were then incubated with 2 mg/mL NHS-PEG4-Biotin (Thermo Scientific, Waltham, MA, USA) in Phosphate-Buffered Saline (PBS), pH 7.4 for 1 h at room temperature with mild agitation, allowing covalent attachment of Biotin to the amino groups via NHS-ester chemistry. Following the reaction, MNs were washed three times with PBS to remove unreacted Biotin. The Biotin-functionalized MNs were then incubated with 10 µg/mL streptavidin (Invitrogen, Carlsbad, CA, USA) in PBS for 1 h at room temperature to form a stable biotin–streptavidin interface. After streptavidin binding, MNs were rinsed gently with PBS to remove excess unbound streptavidin.

Separately, TNF-α capture antibodies (Thermo Scientific, Waltham, MA, USA) were directly biotinylated using a commercial Biotinylation Kit according to the manufacturer’s protocol. The biotinylated antibodies were diluted to 10 µg/mL in PBS, pH 7.4, and incubated with the streptavidin-modified MNs at 37 °C for 1 h to achieve high-affinity immobilization via the biotin-streptavidin interaction. Following incubation, MNs were washed three times with PBS to remove unbound antibodies and stored at 4 °C in PBS until further use. For fluorescence verification of surface modification, biotinylated antibodies were labeled with fluorescein isothiocyanate (FITC) using a molar FITC-to-antibody ratio of approximately 10:1 by incubating at room temperature for 2 h, followed by desalting to remove unreacted FITC. Streptavidin-modified MNs were incubated with FITC-labeled biotinylated antibodies (FITC-Bio-Ab) for 1 h at room temperature, washed with PBS, and imaged using a fluorescence microscope (excitation: 488 nm, emission: 520 nm) to assess modification efficiency.

### 2.3. Evaluation of PMMA Microneedle ELISA Performance

The performance of the functionalized PMMA microneedles was assessed using TNF-α standard solutions at concentrations of 0, 7.81, 31.25, 62.5, 250, and 500 pg/mL. Each MN array was immersed vertically in 100 µL of the corresponding TNF-α solution in a small, flat-bottomed reaction well or microtube and incubated at 37 °C for 1 h with gentle shaking to allow antigen capture. After incubation, the MNs were washed three times with 200 µL PBS (pH 7.4) to remove unbound proteins.

Next, 100 µL of HRP-conjugated detection antibody (1 µg/mL in PBS, pH 7.4) was added, and the MNs were incubated at 37 °C for 30 min. Following three additional PBS washes, 100 µL of 3,3′,5,5′-tetramethylbenzidine (TMB) substrate solution (0.4 mg/mL in citrate–phosphate buffer, pH 5.0) was added directly to the same reaction container, and the enzymatic colorimetric reaction was allowed to proceed for 15 min at room temperature in the dark. After the reaction, 50 µL of 2 M H_2_SO_4_ was added to stop the reaction. The resulting solution was then transferred to a standard 96-well plate for absorbance measurement at 450 nm using a microplate reader (Tecan, Männedorf, Switzerland).

Standard curves were generated by plotting absorbance against TNF-α concentration. The accuracy and reproducibility of the MN-based ELISA were evaluated by comparison with those obtained from commercial ELISA kits, with the linear detection range and correlation coefficient (R^2^) assessed.

### 2.4. Tyramide Signal Amplification Optimization

To enhance assay sensitivity, the effects of using a double the amount of detection antibody in combination with TSA (referred to as double the amount of detection antibody-TSA) were evaluated. Functionalized microneedle arrays were first incubated with TNF-α standard solutions (0, 7.81, 31.25, 62.5, 250, and 500 pg/mL) at 37 °C for 1 h with gentle agitation to allow antigen capture. Following incubation, MNs were washed three times with 200 µL PBS to remove unbound TNF-α.

Next, MNs were incubated with double the standard concentration of HRP-conjugated detection antibody in PBS at 37 °C for 30 min to allow binding to the captured antigen. After three washes with PBS, MNs were incubated with streptavidin-HRP (SA-HRP) at an appropriate concentration for 30 min at 37 °C, followed by three additional PBS washes.

For signal amplification, the TSA system was applied according to the following procedure: MNs were incubated with tyramide working solution at room temperature for 15 min in the dark, allowing HRP to catalyze the formation of reactive radicals that covalently deposited near enzyme-labeled sites. After TSA incubation, MNs were washed thoroughly with PBS to remove unreacted reagents.

The colorimetric readout was then performed by adding TMB substrate solution and hydrogen peroxide to the MN-containing container, allowing the reaction to proceed for 15 min at room temperature in the dark. The reaction was terminated with 50 µL of 2 M H_2_SO_4_, and the solution was transferred to a 96-well plate for measurement of absorbance at 450 nm using a microplate reader.

All experiments were performed in triplicate, and results from standard ELISA, double the amount of detection antibody, and double the amount of detection antibody-TSA conditions were compared to evaluate assay sensitivity, reproducibility, and signal enhancement.

### 2.5. Optimization of Microneedle Insertion Time and Length

To optimize interstitial fluid sampling, MNs with a length of 1.0 mm and identical surface modifications were inserted into fresh porcine skin samples (1 cm × 1 cm) that had been pretreated with TNF-α standard solutions. The MNs were inserted for varying durations (0, 0.5, 1, 5, and 10 min) at room temperature, and gentle pressure was applied to ensure complete skin penetration. After removal, the MNs were gently rinsed with PBS to remove unbound protein. Captured TNF-α was quantified using the previously described ELISA-TSA workflow with double the amount of detection antibody, and the effect of insertion time on capture efficiency and assay sensitivity was determined.

To evaluate the influence of MN length on sampling performance, MNs of different lengths (0.6, 0.8, and 1.0 mm) were inserted into TNF-α-pretreated porcine skin under the previously determined optimal insertion time. Each MN array was incubated under identical conditions to ensure comparability, followed by the same washing and ELISA-TSA detection procedure using double the amount of detection antibody. Absorbance at 450 nm was measured, and the results were analyzed to determine length-dependent differences in antigen capture efficiency and assay sensitivity.

### 2.6. Ex Vivo Detection Using Porcine Skin Models

Fresh porcine skin was obtained from a local abattoir and immediately transported to the laboratory on ice. The skin was carefully trimmed to remove subcutaneous fat and connective tissue and cut into uniform sections of approximately 1 cm × 1 cm. Samples were randomly assigned to three groups: control (untreated), soaking (immersed in 500 pg/mL TNF-α for 2 h at 37 °C), and injection (100 µL of 500 pg/mL TNF-α intradermally). Residual solution was removed, and functionalized microneedle arrays were vertically inserted into each skin section for 10 min at 37 °C to capture interstitial TNF-α.

After removal, the microneedles were processed using the ELISA-TSA workflow with double the amount of detection antibody, and absorbance at 450 nm was measured using a microplate reader to quantify captured TNF-α. Each condition was performed in triplicate to ensure reproducibility.

### 2.7. Cytotoxicity Assessment of PMMA Microneedles

Cytotoxicity assays were performed following previously established protocols [35]. The NIH/3T3 cell line (ATCC CRL-1658, Manassas, VI, USA) was obtained from ATCC. Cells were cultured in Dulbecco’s Modified Eagle Medium (DMEM) supplemented with 10% fetal bovine serum and 1% penicillin–streptomycin (all from Nopu Biosciences, Wuhan) at 37 °C in a humidified incubator with 5% CO_2_ for 24 h to allow cell attachment and growth. After 24 h, MNs with completed surface functionalization were gently placed in direct contact with the cell monolayer. The co-culture was maintained for 3 days under the same incubation conditions, and the culture medium was refreshed every 24 h to maintain optimal cell health.

Cell viability was assessed using Calcein-AM/Propidium Iodide (PI) staining solution (Biyuntian, Beijing, China). MNs and co-cultured cells were incubated with 2 µM Calcein-AM and 4 µM PI in PBS for 30 min at 37 °C in the dark. Fluorescence images were captured using an Olympus IX73 inverted fluorescence microscope equipped with a pHOTOMETRIC iRIS9 sCMOS camera (Olympus, Tokyo, Japan). Pseudo-color images were generated using ImageJ software (ImageJ 1.51k), where green fluorescence indicated live cells and red fluorescence indicated dead cells. The live/dead cell ratio was calculated by counting live (green) and dead (red) cells in at least five randomly selected fields per sample to quantify MN cytotoxicity.

### 2.8. Human-Based Validation Study

Two adult volunteers (ages 25–35) participated in this study. Informed consent was obtained in writing from both participants prior to testing. The study procedures were approved by the Medical Ethics Committee of Harbin Institute of Technology (License No. HIT-2024040) and were conducted in accordance with the guidelines set by the Biosafety Committee of the Center for Life Sciences at Harbin Institute of Technology.

### 2.9. ELISA System Optimization and Multi-Target Detection Validation

Healthy volunteers were recruited to evaluate the in vivo performance of the microneedle-based detection platform. All procedures were approved by the institutional ethics committee, and informed consent was obtained from all participants. MN insertions were conducted under four experimental conditions: (i) blank control without sample loading, (ii) normal control using porcine skin pretreated with PBS, (iii) standard ELISA condition, and (iv) ELISA-TSA system using double the amount of detection antibody. For each condition, MN arrays (1.0 mm length) with identical surface functionalization were gently inserted into the volar forearm skin of healthy volunteers and maintained for 10 min to enable sufficient adsorption of interstitial fluid biomarkers. After removal, MNs were processed following the ELISA-TSA workflow, including sequential incubation with the detection antibody, enzyme conjugate, and chromogenic substrate, followed by colorimetric analysis at 450 nm using a microplate reader.

To further assess the versatility and reliability of the detection platform, serum albumin detection was also performed using the same ELISA-TSA system with double the amount of detection antibody. Calibration curves were first established using porcine skin injected with gradient concentrations of albumin to simulate physiological levels, after which in vivo detection was conducted under identical conditions. The absorbance values obtained from human skin samples were analyzed to evaluate the platform’s sensitivity, reproducibility, and applicability for detecting multiple target biomarkers.

### 2.10. Clinical Validation in an Eczema Patient

A single eczema patient was enrolled to further validate the clinical applicability of the microneedle-based detection system. The experimental design included five groups: blank, control (PBS-injected porcine skin), normal (healthy volunteer), patient healthy skin, and patient affected skin. For each condition, MN arrays were gently inserted into the designated skin site and maintained for 10 min to allow adequate biomarker adsorption, followed by careful removal. Detection of target analytes was then performed using the optimized ELISA–TSA protocol with double the amount of detection antibody, and absorbance was recorded at 450 nm using a microplate reader.

## 3. Results

### 3.1. Fabrication and Mechanical Characterization of PMMA Microneedles

**Figure 2:**
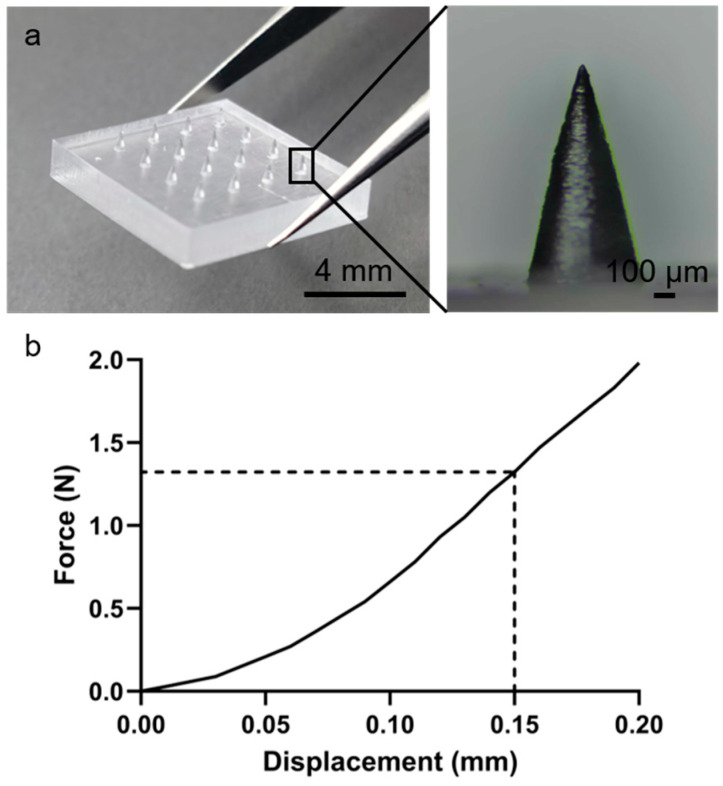
Three-dimensionally printed PMMA microneedles with uniform geometry for skin penetration. (**a**) MN array image. (**b**) Force–displacement curve.

As shown in Figure S1, the 3D printing process sequentially solidified the microneedle model layer by layer, yielding transparent PMMA arrays. Figure 2a demonstrates the successful fabrication of a geometrically uniform 4 × 4 MN array with straight, smooth needles. Mechanical properties were evaluated via axial compression using a universal testing machine (Figure S2). MNs maintained structural integrity under compression, with a maximum load of approximately 0.55 N per needle (Figure 2b), indicating sufficient mechanical strength to penetrate the stratum corneum without deformation. This strength is comparable to previously reported polymer microneedles and is adequate for skin penetration applications [36].

### 3.2. Surface Functionalization and TNF-α Antibody Immobilization

**Figure 3:**
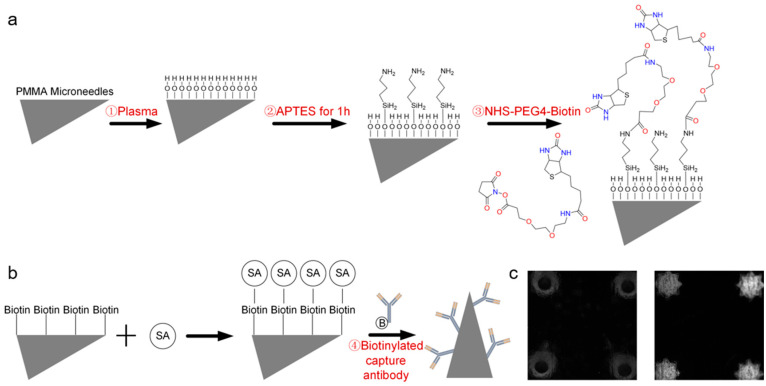
Surface functionalization and TNF-α antibody immobilization. (**a**) Functionalization schematic. (**b**) Immobilization of the biotinylated capture antibody. (**c**) Fluorescence image after modification.

Figure 3 illustrates the surface functionalization strategy and TNF-α antibody immobilization. First, oxygen plasma treatment generated a hydroxyl-rich layer on the MN surface to enhance hydrophilicity and reactivity (Figure 3a). Amino groups were subsequently introduced via APTES silanization, providing reactive sites for covalent conjugation with biotin through NHS-PEG-Biotin chemistry. Figure 3b specifically shows the modification of the biotinylated capture antibodies on the streptavidin-functionalized MN surface, forming a stable and high-affinity Biotin–SA interface for subsequent antibody immobilization.

Fluorescence microscopy confirmed successful functionalization (Figure 3c), with unmodified MNs exhibiting negligible fluorescence and modified MNs showing uniform, intense fluorescence, indicating efficient and homogeneous FITC-labeled TNF-α antibody attachment. This stepwise strategy—plasma activation, silanization, and biotin-streptavidin-mediated capture antibody immobilization—established a stable and highly functional immunorecognition surface suitable for ELISA-based detection. Compared with affinity-based [37] or physical adsorption methods [38], this covalent modification approach offers stronger binding strength, improved antibody orientation, and better retention of bioactivity during washing or incubation. The enhanced surface stability and reproducibility of this functionalization process contribute to the high sensitivity observed in the TSA–ELISA detection results.

### 3.3. Evaluation of PMMA Microneedle Sensor Performance

**Figure 4:**
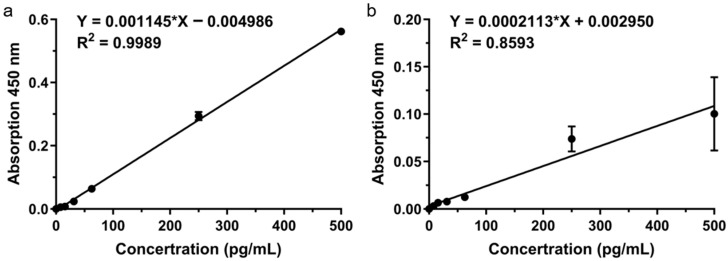
MN-based ELISA sensor performance. (**a**) ELISA standard curve. (**b**) MN sensor linear response.

Although the PMMA microneedle arrays were surface-functionalized, the conventional ELISA system still exhibited limited detection performance for TNF-α [39,40]. As shown in Figure 4b, the MN-based ELISA displayed only a moderate linear correlation across 0–500 pg/mL (R^2^ = 0.8593), which was notably weaker than that of the commercial ELISA standard curve (R^2^ = 0.9989; Figure 4a). The fitted equation, Y = 0.0002113 ∗ X + 0.002950 (C in pg/mL), revealed a relatively low slope and suboptimal linearity, suggesting that despite surface modification, the signal intensity and reproducibility remained insufficient. These findings indicate that the conventional ELISA format on PMMA microneedle substrates is not sensitive enough for precise quantification of cytokines in skin ISF, underscoring the necessity of signal amplification strategies such as TSA enhancement.

### 3.4. Tyramide Signal Amplification Enhancement

**Figure 5:**
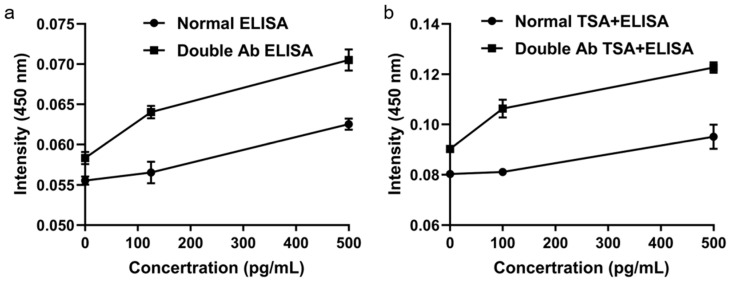
Signal enhancement using tyramide signal amplification. (**a**) Effect of doubled detection antibody. (**b**) Further amplification with TSA.

Doubling the detection antibody concentration under sub-saturation conditions significantly increased the ELISA signal (Figure 5a). Subsequent TSA further enhanced the signal beyond that achieved with either the standard ELISA or the twofold antibody condition alone (Figure 5b), demonstrating that TSA effectively amplifies the enzymatic signal from target binding. The combination of a twofold detection antibody and TSA provided the optimal conditions for high-sensitivity biomarker detection.

### 3.5. Optimization of Microneedle Insertion Time and Length

**Figure 6:**
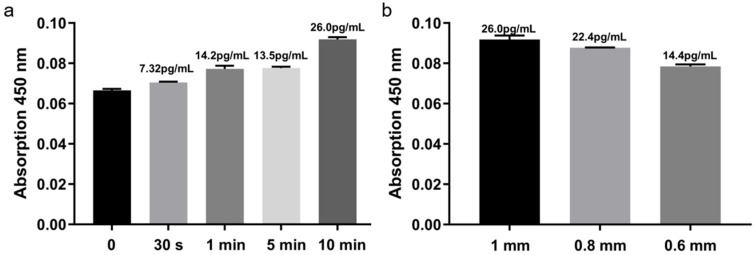
Optimization of microneedle insertion time and length. (**a**) Signal vs. insertion time. (**b**) signal vs. needle length.

Insertion time significantly influenced detection signals (Figure 6a). TNF-α signal gradually increased with prolonged MN residence, indicating effective penetration and target capture. Within 1–5 min, signal increments were modest, suggesting rapid completion of specific binding. At 10 min, the signal nearly doubled compared to 5 min, highlighting enhanced capture efficiency with extended contact. Considering both signal intensity and operational convenience, 10 min was selected as the optimal insertion time.

Needle length also impacted sensitivity (Figure 6b). Longer MNs penetrated deeper into the dermis, accessing more ISF and capturing higher target concentrations. Balancing sensitivity and skin safety, 1.0 mm was established as the standard MN length.

### 3.6. Ex Vivo Detection Using Porcine Skin Models

**Figure 7:**
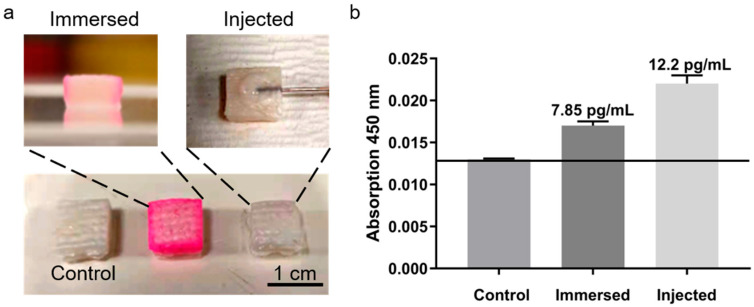
TNF-α detection in porcine skin models. (**a**) Experimental setup. (**b**) Detection signals for different treatments.

Experiments with porcine skin (Figure 7a) revealed significant differences among treatment groups (Figure 7b). TNF-α concentrations were approximately 7.85 pg/mL in the soaking group and 12.2 pg/mL in the injection group, reflecting higher and more uniform dermal distribution after injection, which enhanced detection sensitivity and stability. Therefore, subsequent ISF-based experiments employed subcutaneous injection to ensure consistent signal and reproducibility.

### 3.7. Cytotoxicity Assessment of PMMA Microneedles

**Figure 8:**
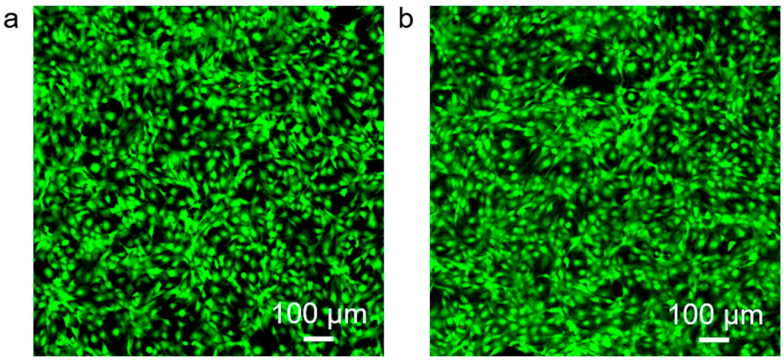
Cytotoxicity assessment using 3T3 fibroblasts. (**a**) Control cells. (**b**) Cells cultured with PMMA microneedles.

Fluorescence microscopy revealed predominantly green fluorescence with minimal red signals in both groups. In the control group (Figure 8a), cells displayed strong green fluorescence with negligible red staining, indicating high viability. Similarly, in the experimental group (Figure 8b), cells maintained comparable fluorescence profiles, with no significant increase in red fluorescence. Morphologically, cells in both groups preserved normal shape and adhesion without evidence of apoptosis or membrane disruption. These observations confirm that MNs exhibit excellent biocompatibility, supporting their safe application for biomarker detection in skin and interstitial fluid under both in vitro and potential in vivo conditions.

### 3.8. Optimization of the ELISA System and Validation for Multi-Target Detection

**Figure 9:**
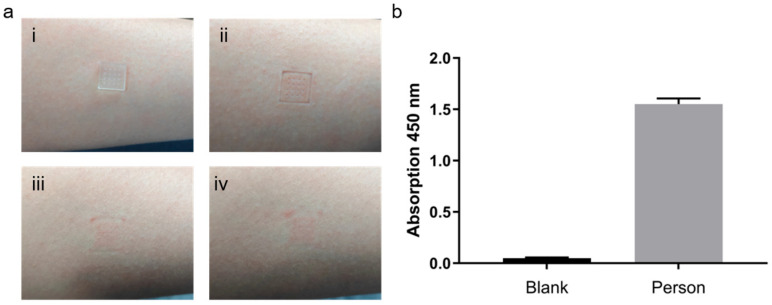
ELISA optimization and multi-target detection in healthy volunteers. (**a**) MN insertion and skin recovery: (**i**) Microneedles inserted into the forearm skin; (**ii**) Micropores formed upon removal; (**iii**) Gradual closure of micropores over time; (**iv**) Skin fully recovered without visible irritation. (**b**) Albumin detection in skin.

Functionalized microneedle arrays were inserted into the forearm skin of healthy volunteers for 10 min to allow adequate adsorption of interstitial biomarkers (Figure 9(ai)). After removal, small and uniformly distributed micropores were visible on the skin surface (Figure 9(aii)). These micropores gradually closed over time (Figure 9(aiii)) and completely healed within approximately 50 min without any observable erythema, edema, or irritation (Figure 9(aiv)), confirming the excellent biocompatibility and minimal invasiveness of the MN platform.

To evaluate the detection performance of the optimized ELISA, MNs were processed according to the standard ELISA workflow after removal. As shown in Figure S3, both the blank and control (PBS-treated) groups exhibited minimal absorbance at 450 nm, indicating negligible nonspecific binding. The standard ELISA condition generated a moderate signal, whereas the configuration employing the TSA system with double the amount of detection antibody produced a substantially stronger and highly reproducible signal. This remarkable enhancement demonstrates that the combination of increased detection antibody concentration and TSA effectively boosts capture efficiency and detection sensitivity, owing to improved antigen–antibody binding kinetics and localized signal amplification on the MN surface.

To further assess the versatility of the optimized TSA system with double the amount of detection antibody, serum albumin was selected as a representative protein biomarker and detected using the same workflow. A well-fitted calibration curve was established using porcine skin spiked with varying albumin concentrations, showing strong linearity within the physiological range (Figure S4). When applied to human skin samples, the MNs successfully captured albumin, yielding distinct absorbance signals compared with the blank control (Figure 9b). These findings confirm that the TSA system with double the amount of detection antibody–based MN–ELISA platform maintains quantitative reliability and broad analyte compatibility, enabling accurate detection of both cytokines and abundant serum proteins.

Collectively, these results indicate that the optimized TSA system with double the amount of detection antibody offers superior analytical performance and biocompatibility, establishing a robust foundation for noninvasive, multi-target, and highly sensitive biomarker monitoring in clinical and point-of-care applications.

### 3.9. Clinical Validation in Eczema Patients

**Figure 10:**
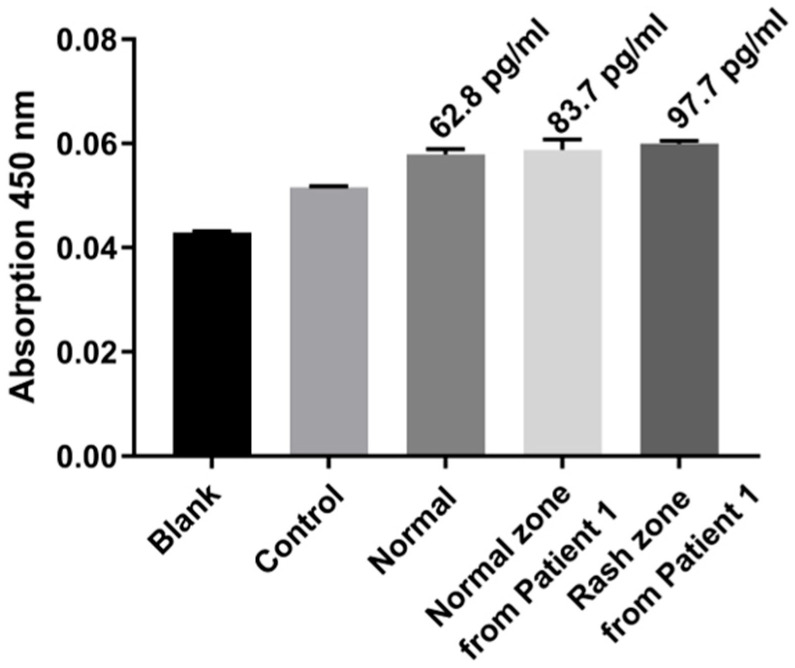
Clinical validation in an eczema patient. TNF-α levels in blank, control, normal, patient healthy, and patient lesion skin.

In eczema patient studies (Figure 10), TNF-α levels exhibited a gradient: blank and PBS control were lowest, normal skin slightly higher, patient healthy region higher, and patient lesion region highest. These results demonstrate that the MN ELISA–TSA platform using double the amount of detection antibody can noninvasively quantify TNF-α, distinguish healthy from diseased skin, and reflect inflammation severity, highlighting its potential for skin disease diagnostics and inflammation monitoring.

### 3.10. TNF-α Levels in Patient Skin: Comparison with Literature

As shown in Table 1, TNF-α levels exhibited a clear gradient from healthy individuals (62.8 pg/mL) to patient healthy regions (83.7 pg/mL) and lesion regions (97.7 pg/mL). Previous studies mostly rely on blood-based measurements, which reflect systemic but not local skin inflammation. In contrast, our MN ELISA–TSA platform samples interstitial fluid directly from the skin, enabling noninvasive, localized, and sensitive detection. This gradient confirms the platform’s ability to distinguish healthy from diseased skin and highlights its potential for precise monitoring of skin inflammation.

**Table 1:** Comparison of TNF-α levels in skin/serum of patients vs. controls.

Study	Sample Type & Region	TNF-α Level	Notes
This study	Patient—healthy skin region	83.7 pg/mL	Non-invasive skin assay, patient unaffected region
This study	Patient—lesion skin region	97.7 pg/mL	Non-invasive skin assay, patient disease region
This study	Healthy individual skin	62.8 pg/mL	Non-invasive skin assay, control healthy skin
Kato et al. [41].	Serum, patients with Lichen planus vs. healthy controls	Patients: 128.20 ± 19.63 pg/mL; Controls: 0.135 ± 0.08 pg/mL	Serum sample, different disease (lichen planus)
Matusiak et al. [42].	Serum, patients with Hidradenitis suppurativa vs. healthy volunteers	Patients: 5.5–35.6 pg/mL; Healthy: <0.1–17.1 pg/mL	Serum sample, range rather than mean
Pirowska et al. [43].	Serum in Psoriasis patients vs. controls	Patients: 1.98 ± 1.50 pg/mL; Controls: 1.13 ± 0.42 pg/mL	Serum sample, lower absolute values, different disease

## 4. Discussion

In this study, we established a 3D-printed PMMA microneedle–based ELISA–TSA platform for the noninvasive quantification of inflammatory biomarkers, including TNF-α and albumin, in subcutaneous interstitial fluid.

The selection of PMMA as the structural material for microneedle fabrication was guided by its unique balance between mechanical robustness, biocompatibility, and optical transparency [44]. Compared with metallic microneedles (e.g., stainless steel, titanium), PMMA offers superior biocompatibility and eliminates concerns of metal ion release or surface corrosion during biological contact. While metal-based microneedles typically exhibit higher mechanical strength, their limited surface modifiability and potential cytotoxicity restrict their suitability for biosensing applications involving delicate immunoassay reactions. Ceramic microneedles [45], on the other hand, provide excellent stiffness and chemical stability but are brittle and difficult to process into complex microscale geometries. In contrast, PMMA can be precisely structured via high-resolution photopolymerization-based 3D printing, allowing reproducible fabrication of sharp and uniform arrays with sufficient strength to penetrate the stratum corneum without fracture. Moreover, its optical clarity facilitates direct visual inspection and integration with optical readout systems, which is advantageous for colorimetric or fluorescence-based biosensing. Therefore, PMMA provides a practical balance of mechanical and functional properties, making it highly suitable for noninvasive detection of inflammatory biomarkers in interstitial fluid.

The platform uses Biotin-SA-Biotin-antibody conjugation for stable antibody immobilization and incorporates TSA to enhance detection sensitivity. Functional validation in both healthy volunteers and eczema patients demonstrated reliable and reproducible results, confirming the platform’s suitability for multi-biomarker analysis. The microneedle approach offers minimal invasiveness, rapid skin recovery, and good safety, making it ideal for routine clinical monitoring.

However, some limitations of the current system should be noted. First, the platform is currently optimized for only a limited set of biomarkers (TNF-α and albumin), and its performance for other analytes remains to be validated. Second, inter-individual variability in skin properties and ISF composition may affect sampling efficiency and quantification accuracy. Third, although the 3D-printed PMMA microneedles are mechanically robust, repeated or long-term use in continuous monitoring scenarios has not yet been evaluated. Finally, integration with portable or wearable readout devices for real-time clinical application requires further engineering optimization.

This microneedle-based system shows great potential for real-time, noninvasive monitoring of inflammation-related biomarkers and could be expanded for other disease applications. It holds promise for personalized healthcare and point-of-care diagnostics. Future studies will focus on enhancing its biomarker detection capabilities and clinical validation.

This microneedle-based system demonstrates considerable potential for real-time, noninvasive monitoring of inflammation-related biomarkers and may be extended to other disease applications. It offers significant promise for personalized healthcare and point-of-care diagnostics. Future studies will focus on improving biomarker detection sensitivity and conducting clinical validation.

## Data Availability

The original contributions presented in this study are included in the article. Further inquiries can be directed to the corresponding authors.

## Supplementary Materials

The following supporting information can be downloaded at: https://www.mdpi.com/article/10.3390/mi16111286/s1: Figure S1, Printing schematic; Figure S2, Compression test setup; Figure S3, TNF-α detection signals under different antibody conditions; and Figure S4, Standard calibration curve for albumin.
