# Supplementary material for: A 3D-Printed PMMA Microneedle-Based TSA-ELISA Platform for Noninvasive Inflammatory Biomarker Detection
Source: Micromachines (Basel). 2025 Nov 14;16(11):1286. doi: 10.3390/mi16111286 (PMC12654171; doi:10.3390/mi16111286)
Supplement: Supplementary file 1 [file micromachines-16-01286-s001.zip › micromachines-3973310-supplementary.pdf]

Supplementary for:
A 3D-Printed PMMA Microneedle-Based TSA-ELISA Platform for Noninvasive Inflammatory Biomarker Detection

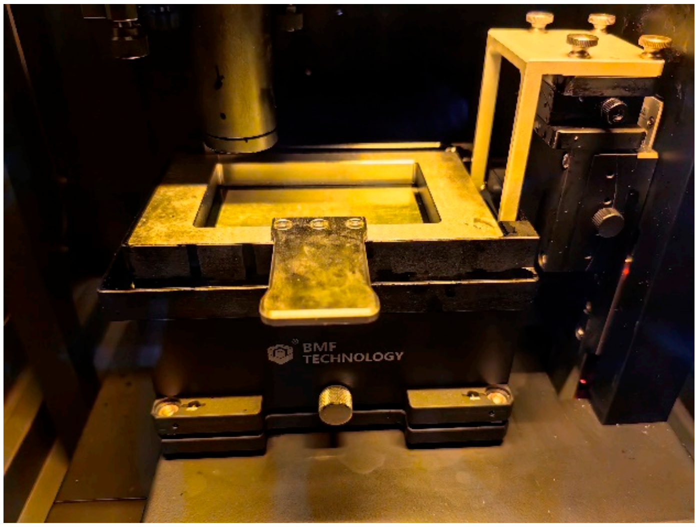

Figure S1. Printing schematic.

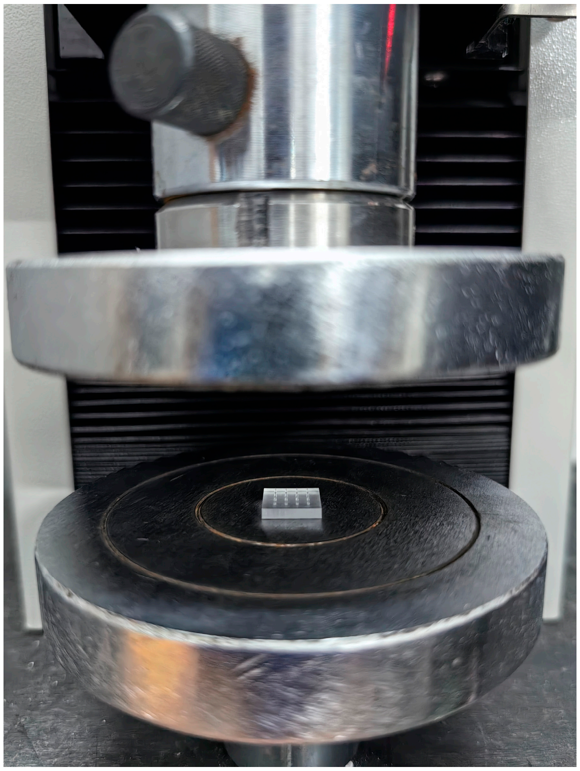

Figure S2. Compression test setup.

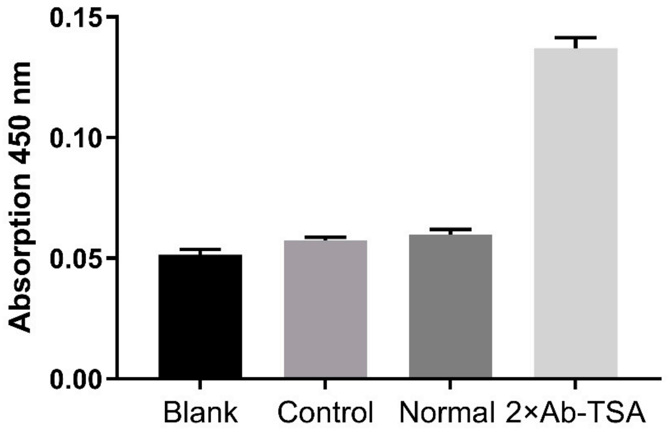

Figure S3. TNF-α detection signals for different antibody conditions.

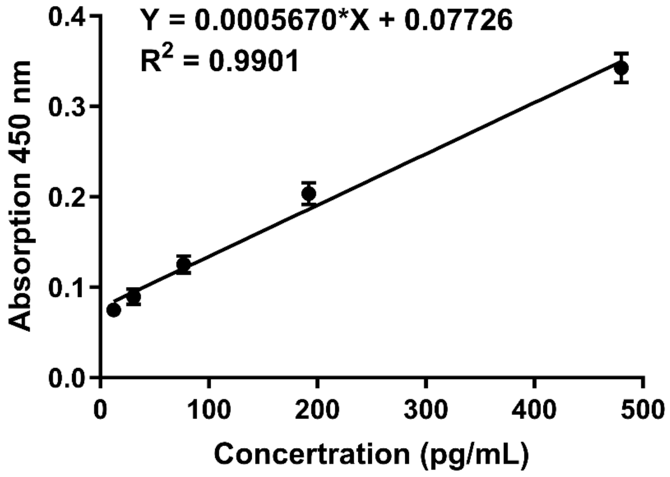

Figure S4. Standard calibration curve for albumin.
